# The impact of achievement motivation on creativity tendency of college students: a moderated mediation model

**DOI:** 10.3389/fpsyg.2025.1720648

**Published:** 2026-01-16

**Authors:** Xiaoming He, Yibo Wang

**Affiliations:** 1 Department of Business Administration, East China University of Science and Technology, Shanghai, China; 2 Ningxia University, Shanghai, China

**Keywords:** achievement motivation, anti-frustration ability, college students, creativity tendency, student competence

## Abstract

**Background:**

With a paradigm shift from “standardized knowledge impartation” to “precision education based on individual traits” in higher education, augmenting college students’ creativity is increasingly emphasized. Understanding the specific impact of motivational factors underlying creativity tendency of college students is attracting scholarly attention in order to propose effective augmenting measures.

**Objective:**

This study focused on the relationship between achievement motivation and creativity tendency of college students, exploring specifically the mediating role of anti- frustration ability and the moderating role of student competence.

**Methods:**

Our sample comprised 1,275 undergraduate students from universities in China. Key constructs in our model (i.e., achievement motivation, anti-frustration ability, student competence, and creativity tendency) were measured by established scales. The PROCESS Macro was adopted to examine this moderated mediation model.

**Results:**

The results showed that (1) Achievement motivation is positively related to creativity tendency of college students; (2) Anti-frustration ability plays a partial mediating role in the relationship; that is, achievement motivation indirectly influences creativity tendency through anti-frustration ability; (3) student competence plays a moderating role in the first stage of the mediation path; that is, the indirect effect of achievement motivation on creativity tendency of college students through anti-frustration ability varies at different levels of student competence and the indirect effect will be greater when student competence is high rather than low.

**Conclusion:**

The results provide insights into how achievement motivation influences creativity tendency of college students, suggesting that universities should enhance creativity cultivation by boosting students’ motivation for achievement and their ability to cope with setbacks. This approach is particularly effective in college students with higher competence.

## 1 Introduction

Creativity has been regarded as ‘the cultural capital of the twenty-first century’ (Sheridan-Rabideau, 2010, p. 54). It is a multifaceted skill shaped both by personal attributes and environmental context (Durnali et al., 2023; Moruzzi, 2021; Said-Metwaly et al., 2017). Key to effective learning in higher education and beyond (Jahnke et al., 2017; Rampersad and Patel, 2014), creativity is an essential skill that encourages college students to cultivate knowledge and competencies within an educational environment by the practices of investigation, collaboration, and synthesis (Livingston, 2010). In the context of higher education, evaluating creativity tendency as a starting point for fostering college students’ creativity reflects a paradigm shift from “standardized knowledge impartation” to “precision education based on individual traits,” which carries profound theoretical foundations and significant practical implications. Hence, it is crucial to understand influencing factors of creativity tendency of college students in order to specifically cultivate and augment their creativity.

Prior research suggests that the most influential factors that affect the creativity development in college students include individual personality (Dweck, 2017; Moruzzi, 2021), motivation (Kalar, 2020; Rodrigues et al., 2019), and capabilities (Caballero-García and Sanchez-Ruiz, 2021), as well as environment (Alencar et al., 2017; Alsharari and Alshurideh, 2020). Given substantial research on these crucial factors that influence creativity development of college students, questions remain that upon entering higher education as freshmen, how to effectively improve these college students’ creativity through differentiated development pathways, based on their heterogeneous levels of creativity tendency. Creativity tendency is a typical psychological trait that includes four descriptors; that is, risk-taking, curiosity, imagination, and challenging. These four attributes of creativity tendency can be commonly perceived in highly creative individuals (Williams, 1980).

Achievement motivation, a core topic in psychological research, constitutes a primary driver of student development. It refers to an internal force for striving for excellence (McClelland, 1961) and pivotal for personal development, with higher levels correlating significantly greater career success (Li and Long, 2017). Yet, despite extensive research in academic (Amrai et al., 2011) and organizational contexts (Lee and Liu, 2009), the exploration of motivational factors underlying creativity tendency, specifically in college students has been scare. As such, as an initial attempt to address those meaningful questions, in this study, we started from deep investigation of achievement motivation. Drawing insights from achievement motivation theory (Atkinson, 1957) and self-determination theory (Deci and Ryan, 1985), we argue that it can promote individuals’ self-efficacy (Li et al., 2023), and encourage innovative thinking and risk-taking (Xu et al., 2025), leading to greater creativity tendency of college students. Moreover, among various types of individual capabilities, anti-frustration ability is more likely to be promoted by achievement motivation (Wu et al., 2023). Hence, we investigated the mediating role of anti-frustration ability, one important type of capacity that can help individuals to bear frustration, grow with it, and fight against it (Yang and He, 2018). Given the unique impact of anti-frustration ability on coping with commonly existing setbacks when initiating innovative solutions (Li et al., 2023), we examined the role of anti-frustration ability and explored the mechanism between achievement motivation and creativity tendency. Further, student competence, defined as essential qualities and critical abilities that facilitate students to adjust to a rapidly changing environment (Wiek et al., 2011), can specifically shape the impact of achievement motivation, because it ensures sustainable resources that can be integrated with the desire for achievement and thus further enhance students’ willingness to endure frustration and encourage innovative problem-solving (Wu et al., 2023). As such, in the context of college students, we examined its moderating role in the relationship between achievement motivation and anti-frustration ability as well as testing a moderated mediation model. Thus, this study examines a moderated mediation model to address the following research questions:

(1) What is the impact of achievement motivation on creativity tendency of college student?
(2) Does anti-frustration ability have any mediating effect on the relationship between achievement motivation and creativity tendency?
(3) What is the role of student competence in the moderated mediation model?

Therefore, this study aims to establish a theoretical foundation, provided new theoretical perspectives, and offer practical guidance on measures and policies of augmenting creativity in college students through higher education.

Testing our hypotheses by a sample of 1,275 undergraduate students from universities in China, we provided supportive evidence. Hence, this study makes several contributions to the literature. First, the investigation of the relationship between achievement motivation and creativity tendency of college students enriches research on the motivational factors underlying creativity tendency. Second, we explored the mechanism of the focal relationship by articulating the role of anti-frustration ability from a positive psychology perspective. Last, in the context of college students, we discussed the moderating role of student competence. Our findings provide a more nuanced understanding of the indirect relationship between achievement motivation and creativity tendency.

## 2 Theoretical background and hypothesis development

### 2.1 Creativity tendency of college students

Creativity tendency is a specific psychological trait that indicates individuals’ willingness and intention to be creative in problem solving (Wu, 2025). Highly creative people commonly demonstrate four traits (i.e., risk-taking, curiosity, imagination, and challenging; Williams, 1980). These psychological traits or inclinations toward creativity are indicative of an individual’s affective creativity (Weiss et al., 2021). Individuals who possess stronger creativity tendencies are considered more proficient in employing their creativity, in contrast to those lacking such tendencies (Lin and Wang, 1994). Further, an individual’s creative tendency can shape their effectiveness in tasks requiring creative problem-solving (Hsiao, 2017). Findings of reviewing prior research suggest that the most influential factors that affect creativity development of college students include personality, motivation, learning activities, and environment (Correa and Mourao, 2025).

First, consistent with prior research on creativity (Dweck, 2017; Moruzzi, 2021), studies on specific attributes of individual personality suggest that certain individual traits can improve a student’s ability to produce novel ideas and solutions. For instance, students who exhibit a higher degree of openness to experience are typically more amenable to new information and flexible in their thinking, thereby enabling greater creative expression in their academic disciplines (Correa and Mourao, 2025; Lizarraga et al., 2014). Second, intrinsic motivation attributes, such as entrepreneurial intention and achievement motivation, encourage the cultivation of an innovative mindset in students through a pedagogy of experimentation and iterative learning. By engaging in this process, which involves testing ideas and learning from setbacks, students enhance their creativity through direct exploration (Kalar, 2020; Rodrigues et al., 2019).

Third, student learning activities improve students’ competence and subsequent academic performance. By promoting engagement in interactive formats like collaborative projects and hands-on tasks, these activities boost student motivation, strengthen capacities, and further promote their creativity (Caballero-García and Sanchez-Ruiz, 2021). Finally, a key role of higher education is to prepare individuals for the workforce by fostering autonomy, openness, and flexibility (Alencar et al., 2017; Alsharari and Alshurideh, 2020; Egan et al., 2017). It is now widely acknowledged that creativity development of college students can be stimulated by teachers and teaching environment (Correa and Mourao, 2025; Gao, 2023; Marczewska et al., 2024). By establishing an open, supportive, and challenging environment, higher education can enhance student motivation to explore new ideas, think critically, and proactively develop innovative solutions (Egan et al., 2017; Gao, 2023).

Because creativity tendency is a typical creative trait, this study focuses on the impact of a specific type of psychological traits, namely achievement motivation, among the four categories of influential factors. Extensive research has been done on the influence of achievement motivation in academic (Amrai et al., 2011; Li et al., 2023), organizational (Lee and Liu, 2009), and social contexts (Xu et al., 2025). For instance, Li et al. (2023) explored the positive relationship between achievement motivation and self-efficacy of college students, which is mediated by perceived social support. Moreover, Lee and Liu (2009) identified that achievement motivation is positively related to employees’ work attitude through psychological contracts. Further, Xu et al. (2025) found that through the mechanism of creativity, achievement motivation promotes performance of wargame players. Nevertheless, how such motivational factor affects creativity tendency of college students has been under investigation. As such, this study starts by exploring the influence of achievement motivation on creativity tendency.

### 2.2 The relationship between achievement motivation and creativity tendency

As a typical creative trait, creativity tendency is commonly observed in individuals with high creativity and assessed by four dimensions (i.e., risk-taking, curiosity, imagination, and challenging; Williams, 1980). Specifically, risk-taking reflects an adventurous spirit—such individuals stand by their ideas and confront the unknown through experimentation, even in the face of potential criticism or failure (Gostoli et al., 2017). Curiosity, as a central driver of creativity, motivates people to question assumptions and explore underlying truths. Imagination enables the concretization of diverse thoughts, often transcending reality to explore boundless possibilities. Lastly, challenging indicates a preference for complexity, with which individuals are able to employ logical reasoning to identify solutions amid chaotic situations and address problems in a structured way (Wu, 2024). Empirical studies further suggests that, while high creativity tendency promotes innovative problem solving (Hsiao, 2017), creative tendency is affected by various personality traits, including openness, extroversion (Chen et al., 2023), anxiety (Chiu et al., 2019), optimism, and emotional exhaustion (Li Y. et al., 2019).

In this study, we focused on a typical type of intrinsic motivation of creativity, achievement motivation (Correa and Mourao, 2025), and investigated the impact of achievement motivation on creative tendency of college students, given its important role in their development (Li et al., 2023). Achievement motivation is an internal drive for success and achievement (Orakci, 2023). It represents an individual’s desire to engage in tasks that are personally meaningful and demanding, striving for outstanding performance and superior outcomes, as well as the aspiration to excel beyond others (McClelland, 1961). According to achievement motivation theory (Atkinson, 1957), individual actions are guided by the dynamic opposition of two motivational tendencies: the pursuit of success (PS) and the avoidance of failure (AF). PS constitutes a dispositional tendency to approach tasks that offer mastery experiences, driven by the positive affect of pride associated with accomplishment. AF, conversely, constitutes a predisposition to avoid tasks in which failure is probable, motivated by the desire to evade the negative affect of shame (Xu et al., 2025). Moreover, self-determination theory posits that achievement motivation, as a key type of intrinsic motivation, serves as a powerful catalyst for heightened creative performance by driving personal interest and a sense of autonomy in the task itself (Deci and Ryan, 1985). Thus, we argue for a positive impact of achievement motivation on creativity tendency of college students.

Individuals with high levels of achievement motivation exhibit a stronger desire for success and are more likely to attain it compared to those with lower motivation (McClelland, 1961). With high achievement motivation, college students may be more willing to take risks, be curious of things, and prefer to cope with complex situation by imagination, thus leading to a high level of creativity tendency. First, with high achievement motivation, college students may engage more in tasks with enhanced goal commitment (Hewitt and Flett, 1991; McClelland, 1985). As such, they are liable to take risks, even in the face of potential failure. Moreover, motivated by pursuit of success and avoidance of failure, they may challenge assumptions and explore diverse ideas through imagination. Further, they may allocate greater cognitive resources to high-risk tasks and search for creative solutions. As such, college students with strong achievement motivation tend to exhibit a higher creativity tendency, whereas those who lack achievement motivation and tend to avoid failure are likely to demonstrate a weaker creativity tendency. We thus propose our first hypothesis that: Achievement motivation positively influences creativity tendency of college students (H1).

### 2.3 The mediating role of anti-frustration ability

The notion of anti-frustration ability originates from frustration tolerance (Yang and He, 2018). Nevertheless, unlike frustration tolerance, which refers to the capacity to withstand pressure in the face of setbacks, anti-frustration ability refers to the capacity that can help individuals tolerate frustration, grow with it, and proactively address it (Yang and He, 2018). From the perspective of positive psychology, prior research suggests that commonly tolerant of frustration, individuals with high resilience are generally considered having high anti-frustration ability and more included to experience positive emotion, even when encountering difficult situations (Fredrickson, 2003). With strong anti-frustration ability, individuals are more liable to have strong confidence when perform tasks, more tolerant to frustration, psychologically prepared to understand and address frustrated situations with interpersonal interaction ability (Zhang, 2013). As such, with strong anti-frustration ability, individuals can endure stressors and endeavor to overcome them (Li Q, et al., 2019).

On the one hand, those with strong achievement motivation demonstrate greater persistence and motivation when confronting adversities. Students who possess this motivational trait are thus able to persevere in difficult environments and overcome various academic obstacles (Li et al., 2023). As such, strong achievement motivation improves anti-frustration ability of college students. On the other hand, when confronted with frustration, individuals with high anti-frustration ability utilize various strategies like self-deprecation or humor to foster positive emotions, reduce stress, and adapt to environmental demands (Block and Kremen, 1996; Tugade and Fredrickson, 2004). According to the broaden-and-build theory (Fredrickson, 2013), positive emotions facilitate the expansion of attentional, cognitive, and behavioral repertoires, enhancing one’s capacity to perceive the environment with greater acuity and to process pertinent information more effectively in challenging circumstances (Contractor et al., 2021). Equipped with stronger confidence and better psychologically prepared, college students can leverage their broadened attention and cognition to behave more adventurously and better cope with complex situations. Hence, owing high anti-frustration ability enables college students to be more likely to be creative. This suggests that college students with strong anti-frustration ability tend to exhibit a higher level of creativity tendency, whereas those lacking such abilities—who fail to confront setbacks appropriately and adopt rational coping strategies—are likely to demonstrate a lower level of creativity tendency. Taken together, achievement motivation enhances anti-frustration ability and such ability, in turn, facilitates the development of creativity tendency of college students. Therefore, we propose our second hypothesis to examine the indirect positive relationship: anti-frustration ability mediates the relationship between achievement motivation and creativity tendency of college students (H2).

### 2.4 The moderated mediation effect of student competence

Student competence is considered a vital and enduring resource that can shape the impact of achievement motivation. Student competence, namely core competence of a student, refers to the fundamental qualities and key abilities that allow students to adapt to a dynamic global environment (Wiek et al., 2011). Student competence promotes the growth of creativity, self-direction, and self-motivation, and thus assists students in overcoming diverse challenges (Wu et al., 2023). Scholars often view student competence as a central element that enhances a student’s capacity to adapt to the changing environment (Lin, 2016). The theoretical foundation of core competencies is multifaceted and interdisciplinary, including cognitive psychology theory that explains its cognitive processing nature (Neisser, 1967) and sociocultural theory that explains its social interaction nature (Vygotsky, 1978). Among studies on student competence, for example, Huang et al. (2016) discussed a multi-faceted conceptualization, encompassing self-related literacy (e.g., learning skills, mental health, self-management), culture-related literacy (e.g., language, mathematics, science), and society-related literacy (e.g., social responsibility, value, beliefs). Similarly, Guerra and Bradshaw (2008) identified five facets of core competencies that young people should develop: (1) a positive self-identity, (2) self-control, (3) decision-making skills, (4) a moral belief system, and (5) prosocial connectedness. Fostering college students’ core competence is a crucial objective for the substantive development of higher education. It also represents a principal approach to fulfilling the fundamental mission of moral education and talent development (Lin, 2016).

College students with high achievement motivation can be persistent and motivated to address adverse situations (Li et al., 2023). However, for instance, without the support of student competence, they may not be able to analyze the confronted issues by the use of rationale approaches and eventually fail to figure out feasible solutions. In contrast, with strong competence, college students have a solid literacy basis to cope with environmental dynamics (Wiek et al., 2011). Those with achievement motivation may leverage their decision-making skills to mitigate external uncertainties that may trigger frustrated conditions. They may also use their interaction skills to understand those complex situations when aiming to address them. Eventually, equipped with this vital resource, students with strong achievement motivation can be more confident to tolerate difficult situations, addresses them and grow with them. As such, students’ anti-frustration ability can be further improved, which in turn improves their creativity tendency. We thus propose our third hypothesis: student competence will moderate the indirect relationship in the way that it strengthens the positive impact of achievement motivation on anti-frustration ability, such that the indirect effect is greater when student competence is high rather than low (H3).

**Figure 1:**
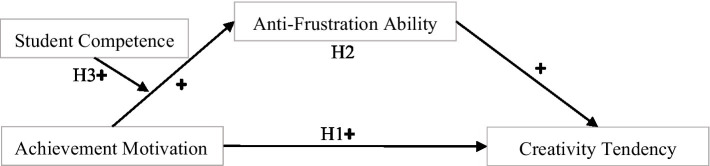
Theoretical framework.

In sum, we established a moderated mediation model (see Figure 1) in which achievement motivation influences creativity tendency of college students through the mediating role of anti-frustration ability, while student competence moderates the first stage of the indirect relationship between achievement motivation and creativity tendency of college students. As such, this model provides a theoretical framework to investigate the mediating (anti-frustration ability) and moderating (student competence) mechanisms of achievement motivation in explaining creativity tendency of college students. This model thus offers a heuristic framework on how and when creativity tendency of college students’ is influenced by their achievement motivation.

## 3 Materials and methods

### 3.1 Participants

We sent out online questionnaires via *Questionnaire Star* to freshmen in six universities in Beijing, Shanghai, Chengdu, and Guangzhou. These representative universities were selected from the eastern, western, southern, and northern regions where the research team had established connections to ensure geographic diversity and facilitate access. An informed consent form was required to be signed before filling out the questionnaire. Because freshmen have not experienced university life, their creativity is relatively malleable. Understanding the creative tendencies of college freshmen, targeted and appropriate training models can be developed to fully leverage the positive influence of higher education and stimulate their creative potential along their university life. As such, investigating freshmen becomes especially suitable for this study. Thus, freshmen were selected for our investigation in this study. We received 1,556 questionnaires and after eliminating responses with missing data, we finally obtained a total of 1,275 valid questionnaires with a recovery rate of 82%, including 571 boys (44.8%) and 704 girls (55.2%).

### 3.2 Measures

#### 3.2.1 Creativity tendency scale

This study used the creativity tendency scale in traditional Chinese proposed by Lin and Wang (1994), which is based on Williams (1980)’s creativity assessment packet. Four common dimensions were identified to evaluate creativity tendency; that is, risk-taking, curiosity, imagination, and challenging (Williams, 1980). The scale comprises 50 items (See online Appendix B). Respondents rate each item using a 3-point Likert scale, and a high total score suggests strong creativity tendency. Internal consistent coefficients for the four dimensions are 0.61, 0.74, 0.67, 0.74, respectively, and the whole scale’s Cronbach’s *α* is 0.89, indicating good validity and reliability.

#### 3.2.2 Achievement motivation scale

The initial achievement motivation scale was developed by Norwegian psychologists T. Gjesme and R. Nygard in 1970. The Chinese version was jointly translated in 1988 and revised in 1992 by Chinese scholar R. Ye and Norwegian scholar K. A. Hagtvet (Ye and Hagtvet, 1992). This scale has a total of 30 items and includes two dimensions; that is, motivation to avoid failure and motivation to pursue success (See online Appendix B). Using a 5-point Likert scale, respondents were required to rate each item. The total score of pursuit of success is Mps and the total score of avoidance of failure is Maf. As such, the score of the whole scale is MA = Mps-Maf. A high score suggests strong achievement motivation of an individual. The internal consistency coefficient for motivation to pursue success (items 1–15) is 0.86 and the internal consistency coefficient for motivation to avoid failure (items 16–30) is 0.79. In this study, the whole scale’s Cronbach’s *α* is 0.83.

#### 3.2.3 Anti-frustration ability scale

This study used a total of 48 items proposed by Zhang (2013) to measure anti-frustration ability (See online Appendix B). This scale comprises 10 factors, including frustration tolerance, confidence, frustration experience, frustration resilience, frustration cognition, interpersonal interaction ability, career programming ability, willpower, psychological preparation, and attribution ability. We used a 5-point Likert scale for this variable in this study. A high total score suggests a high level of anti-frustration ability. Internal consistent coefficients for the 10 factors range from 0.71 to 0.84. And the whole scale’s Cronbach’s *α* in this study is 0.90.

#### 3.2.4 Student competence scale

This study used a total of 43 items proposed to measure student competence (See online Appendix B). This scale is based on the scale for students’ core competence developed by Lin (2017) and further revised by Zhang (2020). This scale comprises nine factors, including national identity, global understanding, esthetic sentiment, literacy achievement, problem-solving, rational thinking, self-reflection, self-management, and enjoyment of learning. Using a 5-point Likert scale, respondents were required to rate each item. A high total score indicates a high core competence of college students. Internal consistent coefficients for the nine factors range from 0.68 to 0.91. And Cronbach’s *α* for the whole scale in this study is 0.92.

### 3.3 Common method variance

A confirmatory factor analysis was conducted in this study to assess the model fit (see Table 1). The model fit indices are important to evaluate the level of alignment between the data and the model. Moreover, this study examined the potential of common method biases by the Harman’s single-factor test. An unroted exploratory factor analysis showed that four factors had an eigenvalue higher than 1. Further, the first factor explained 21.52% of the total variance, well below the cutoff value of 40% (Podsakoff et al., 2003). As shown in Table 1, when we placed all items on a single factor, the one-factor model was poorly fitted (χ^2^/df = 9.381, CFI = 0.517, TLI = 0.362, RMSEA = 0.238, SRMR = 0.197) and thus we concluded that the model cannot be accepted. When we assigned all the items by the proposed model, the model fit was good (χ^2^/df = 2.029, CFI = 0.961, TLI = 0.912, RMSEA = 0.049, SRMR = 0.040). Based on these results, we concluded that no serious common method biases were identified in this study.

**Table 1:** Results of confirmatory factor analysis.

Model	χ^2^/df	CFI	TLI	RMSEA	RMSR
four-factor model	2.029	0.961	0.912	0.049	0.040
three-factor model	3.717	0.831	0.736	0.113	0.102
two-factor model	6.310	0.643	0.512	0.169	0.146
single-factor model	9.381	0.517	0.362	0.238	0.197

The combinations were as follows: (1) Three-factor model: a combination of achievement motivation and student competence; (2) Two-factor model: a combination of achievement motivation, anti-frustration ability and student competence; (3) Single-factor model: a combination of all variables.

### 3.4 Reliability and validity

We evaluated reliability and validity of our constructs by various methods. First, we conducted reliability analyses for all constructs. As shown in online Appendix A, Cronbach’s *α*, McDonald’s *ω* and the composite reliabilities (CR) of all constructs are above 0.60, indicating adequate internal reliability of the constructs (Bagozzi and Yi, 1988). Second, validity analyses were conducted for each construct. Following Fornell and Larcker (1981), a factor-loading method were adopted to test the convergent validity. All the factor loadings are above 0.60, indicating adequate convergent validity. Further, to evaluate discriminant validity, an average variance extracted (AVE) method was adopted. AVE values of all constructs are shown in online Appendix A. The square roots of the AVE for each construct are higher than the correlations, indicating adequate discriminant validity (Fornell and Larcker, 1981).

### 3.5 Data analysis

In this study, we took several specific steps to conduct data analysis. First, AMOS26.0 was used to conduct the confirmatory factor analysis and validate the measurement model. Second, with validated data, SPSS 27.0 was used to conduct the descriptive and correlation analysis. Third, both the mediation test and the moderated mediation test were conducted by the use of the PROCESS Macro (Hayes, 2017). As an extension of the Sobel test (Sobel, 1982), this bootstrapping-based mediation test procedure has been encouraged to be applied over alternative procedures (e.g., Baron and Kenny, 1986; Li et al., 2023; Wu et al., 2023; Yam et al., 2018). This procedure does not require for a normal sampling distribution of indirect effects, and results of simulation studies have approved that it is more powerful and valid than traditional statistical methods (MacKinnon et al., 2004).

Specifically, Model 4 of the PROCESS Macro is designed to examine a mediation effect, which included a set of bootstrapping tests with 5,000 resamples to produce a 95 percent confidence interval (CI) around the estimated direct and indirect effects. The results indicate the relative direct effect (*c*) of X on Y and the relative the indirect effect (*a* * *b*) through M (i.e., *a* the path coefficient X → M and *b* the path coefficient M → Y). Model 7 of the PROCESS Macro is specifically designed to test a first-stage moderated mediation effect, where conditional indirect effect equals (*a_1_* + a_3_*W)*b and *a_3_* is the path coefficient of the interaction between X and the moderator W (Hayes, 2017). In this study, we focused on Model 7 to examine both the mediation effect of anti-frustration ability and the moderated mediation effect of student competence, because when the moderator W is at the mean value, the simple mediation effect can be examined.

## 4 Results

### 4.1 Descriptive statistics and correlations

Table 2 presented the descriptive statistics, specifically the means and standard deviations of the four variables, and correlations among these variables. As shown in Table 2, all the three variables are positively correlated with creativity tendency (*p* < 0.001) and both achievement motivation and student competence are positively correlated with anti-frustration ability (*p* < 0.001).

**Table 2:** Descriptive statistics and correlations.

Variables	Mean	S. D.	1	2	3	4
Creativity tendency	2.23	0.25	1			
Achievement motivation	0.06	0.96	0.45***	1		
Anti-frustration ability	3.28	0.63	0.50***	0.25***	1	
Student competence	3.21	0.79	0.17***	0.22***	0.21***	1

*N* = 1,575; *p < 0.05, **p < 0.01, ***p < 0.001.

### 4.2 The relationship between achievement motivation and creativity tendency

H1 predicts that achievement motivation positively influences creativity tendency of college students. The results are presented in Model 1 of Table 3, which show that the coefficient of achievement motivation was positive and statistically significant (coefficient = 0.19, *p* = 0.000), suggesting a positive association between achievement motivation and creativity tendency. Meanwhile, results also show that the total effect of achievement motivation on creativity tendency is 0.19. We concluded that H1 receives support.

**Table 3:** Testing for the mediation effect of anti-frustration ability.

Variables	Model 1 DV: Creativity tendency		Model 2 DV: Anti-frustration ability		Model 3 DV: Creativity tendency	
	coefficient	*t*	coefficient	*t*	coefficient	*t*
Achievement motivation	0.19	3.56***	0.17	5.43***	0.16	2.98**
Anti-frustration ability					0.20	4.08***
*R^2^*	0.13		0.15		0.22	
*F*	128.68***		108.62***		190.01***	

*p < 0.05, **p < 0.01, ***p < 0.001.

### 4.3 The mediating role of anti-frustration ability

**Figure 2:**
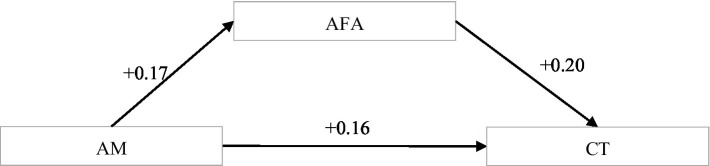
AFA as the mediator between AM and CT. AFA, Anti-Frustration Ability; AM, Achievement Motivation; CT, Creativity Tendency.

H2 predicts that anti-frustration ability plays a mediating role in the relationship between achievement motivation and creativity tendency of college students. We followed Hayes (2017) to test this partial mediation and results are presented in Models 2 and 3 of Table 3. As shown in Model 2, achievement motivation was positively related to anti-frustration ability (coefficient = 0.17, *p* = 0.000). In Model 3, anti-frustration ability was significantly related to creativity tendency (coefficient = 0.20, p = 0.000). Also shown in Model 3, when the effect of anti-frustration ability was controlled, the effect of achievement motivation on creativity tendency was positive and significant (coefficient = 0.16, *p* = 0.003). The mediating model is shown in Figure 2.

Consistent with these results, both the direct effect (0.16, *p* = 0.003) and the indirect effect were significant (0.03, *p* = 0.000; see Table 4). Taken together, these results supported that anti-frustration ability plays a mediating role in the relationship between achievement motivation and creativity tendency of college students. Therefore, H2 was supported.

**Table 4:** Mediation model effect.

Effect type	Effect size	Boot SE	95% CI
Total effect	0.19	0.05	[0.10, 0.28]
Direct effect	0.16	0.05	[0.07, 0.26]
Indirect effect	0.03	0.01	[0.01, 0.05]

### 4.4 The moderated mediation effect of student competence

**Figure 3:**
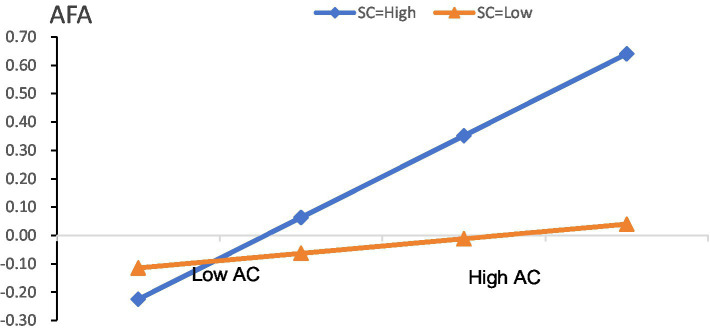
SC as the moderator for the AC-AFA. AFA, anti-frustration ability; AC, achievement motivation; SC, student competence.

Next, we tested H3 with a moderated mediation model (Edwards and Lambert, 2007). This test was also implemented using the PROCESS macro Hayes (2017), and the results are presented in Models 1 and 2 of Table 5. As shown in Model 1, the interaction coefficient of achievement motivation and student competence was positive and significant (coefficient = 0.15, *p* = 0.004). We conducted a simple slope analysis and presented the results in Table 6. As shown in Table 6, when student competence was high (M + 1SD), achievement motivation was significantly and positively related to anti-frustration ability (coefficient = 0.29, *p* = 0.000) while the coefficient was not significant though positive (coefficient = 0.05, n.s.) when student competence was low (M-1SD). The simple slope difference test also returned significant results (*p* = 0.000). We further plotted this moderating effect in Figure 3. As shown in Figure 3, creativity tendency of college students increased with achievement motivation at a faster rate when student competence was higher.

**Table 5:** Testing for the moderating effect of student competence.

Variables	Model 1 DV: Anti-frustration ability		Model 2 DV: Creativity tendency	
	Coefficient	*t*	Coefficient	*t*
Achievement motivation	0.17	6.82***	0.16	2.98**
Anti-frustration ability			0.20	4.08***
Student competence	0.08	1.99*		
Achievement motivation × Student competence	0.15	2.87**		
*R^2^*	0.19		0.22	
*F*	132.09***		190.01***	

*p < 0.05, **p < 0.01, ***p < 0.001.

**Table 6:** The moderating effects at different levels of student competence.

Student competence	Coefficient	Boot SE	95% CI
−0.79 (Mean–1SD)	0.05	0.03	[−0.02, 0.10]
0 (Mean)	0.17	0.07	[0.04, 0.29]
0.79 (Mean + 1SD)	0.29	0.10	[0.08, 0.47]

Further, we estimated the conditional indirect effect using a bootstrapping procedure. Table 7 provides further evidence of the conditional indirect effect. As shown in Table 7, the indirect effect between achievement motivation and creativity tendency mediated by anti-frustration ability was positive and significant when student competence was high at mean plus one standard deviation (0.06, *p* = 0.000). However, this conditional indirect effect was also significant when student competence was at its mean value (0.03, *p* = 0.003) but lost its significance when student competence was at a low level (mean minus one standard deviation; 0.01, n.s.). Consistent with the results from Models 1 and 2 of Table 5, these results demonstrate that the hypothesized indirect effect was conditional on the presence of student competence. Taken together, these results supported H3 that student competence moderates the indirect relationship in the way that it strengthens the positive relationship between achievement motivation and anti-frustration ability, such that the indirect effect is greater when student competence is high rather than low.

**Table 7:** Conditional indirect effects.

Student competence	Effect size	Boot SE	95% CI
−0.79 (Mean–1SD)	0.01	0.01	[−0.01, 0.03]
0 (Mean)	0.03	0.01	[0.01, 0.05]
0.79 (Mean + 1SD)	0.06	0.01	[0.03, 0.08]

## 5 Discussion

An emphasis on cultivating college students’ creativity in the current era reflects a paradigm shift from “standardized knowledge impartation” to “precision education based on individual traits.” As such, evaluating creativity tendency of college students and its influential factors can be an important starting point for fostering their creativity through higher education. Nevertheless, given the importance of motivational factors of college students underlying creativity tendency, the impact of specific types of motivation is still under explored (Amrai et al., 2011; Correa and Mourao, 2025). This study focused on achievement motivation and investigated its impact on creativity tendency of college students. Our empirical findings indicated that while achievement motivation promotes creativity tendency of college students, anti-frustration ability mediates the relationship and student competence plays a moderating role such that it strengthens the impact of achievement motivation on anti-frustration ability, which in turn further enhances creativity tendency. Thus, this study provides profound theoretical foundations and significant practical implications to higher education institutions and teachers in high education on how to enhance creativity of college students by guiding the development of specific individual psychological and behavioral aspects.

### 5.1 The relationship between achievement motivation and creativity tendency of college students

Our findings indicate that achievement motivation is positively related to creativity tendency of college students, providing support to our first hypothesis. The motivation to achieve success or to avoid failure constitutes a psychological force that enables individuals to successfully complete tasks and accomplish their goals due to their self-confidence, self-identity, and the need for achievement (McClelland, 1961). An individual’s belief in their capability to complete a specific task fosters a proper understanding of confronted complex situations. Such self-belief is also a prerequisite for making an informed judgment about their confidence in successfully completing the task. An internal need for achievement can also drive high commitment and engagement, where pursuing ambitious goals refines one’s ability judgments (Mei et al., 2019). As such, individuals are more willing to take risks curiously and prefer to cope with complex scenarios with imagination, thereby enhancing creativity tendency.

Prior research has largely examined the impact of achievement motivation on individual psychological traits, behaviors and outcomes. Scholarly studies provide evidence for a positive impact of achievement motivation on various types of performance, such as performance in wargames (Xu et al., 2025) and academic achievement (Steinmayr et al., 2019). It also influences individual psychological traits like self-efficacy (Li et al., 2023) and behaviors like student engagement (De Castella et al., 2013). However, the relationship between achievement motivation and creativity of college students has not been adequately examined (see Correa and Mourao, 2025 for a thorough review). Hence, our investigation not only complements the research on achievement motivation but also enriches the exploration of motivational factors underlying creativity tendency of college students.

Our investigation also suggests that higher education institutions and teachers should acknowledge the importance of psychological drivers like achievement motivation to creativity. By consciously designing curricula that present calibrated challenges, assessments that reward original processes, structures that enable safe experimentation, and a culture that celebrates the creative journey, higher education institutions can transform achievement motivation from a force that merely drives grade competition into a powerful engine for developing innovative capacity. This encourages initiatives to establishing an educational environment where the most motivated path to achievement is, by design, a creative one.

### 5.2 The mediating role of anti-frustration ability

In this study, we found that an indirect effect of achievement motivation on creativity tendency exists through the mediating factor of anti-frustration ability of college students, supporting our second hypothesis. As such, a mediation model “achievement motivation → anti-frustration ability → creativity tendency” was constructed to support a partial mediation effect of anti-frustration ability, in which achievement motivation was still significantly related to creativity tendency.

Anti-frustration ability denotes the capacity not merely to endure adversity but to grow from it and proactively engage with it (Ou et al., 2013). Prior research suggests that individuals with strong achievement motivation exhibit heightened persistence and drive in the face of adversity. Consequently, students with this psychological trait can persevere in challenging environments and navigate through various academic obstacles, leading to improved anti-frustration ability (Yang and He, 2018). Moreover, research indicates that with high anti-frustration ability, individuals exhibit greater frustration tolerance and a heightened propensity for positive feelings, even in challenging circumstances (Fredrickson, 2003). Such individuals tend to approach tasks with stronger self-confidence, greater persistence, and the psychological readiness to navigate setbacks, often leveraging well-developed interpersonal skills (Zhang, 2013). Fredrickson (2003) stated that since positive emotions may broadened individuals’ attention and cognition, college students can employ their expanded attentional and cognitive resources to act more venturesomely and navigate complex situations effectively. Thus, possessing strong anti-frustration ability makes students more likely to demonstrate creativity.

In sum, our findings of testing this mediation model suggest a feasible pathway whereby achievement motivation is transmitted to creativity tendency via anti-frustration ability. Hence, this study contributes to the research on the impact of motivational factors on creativity tendency by opening the black box and proposing an explainable mechanism. Although prior research has investigated various types of individual capabilities underlying creativity (Correa and Mourao, 2025), among them, anti-frustration ability is more likely to be promoted by achievement motivation (Li et al., 2023). As such, the investigation of anti-frustration ability as the mediator complements the research on the relationship between achievement motivation and creativity tendency.

The cultivation of anti-frustration ability becomes the master key, unlocking the creative potential latent in students’ achievement motivation. With this understanding, higher education institutions and teachers should be aware that efforts should be taken to cultivate students’ anti-frustration ability, which can enhance their creativity tendency. Moreover, in order to improve their anti-frustration ability, they may take measures to augment college students’ motivation for pursuit of success and avoidance of failure. For instance, the institutions may proactively strengthens students’ anti-frustration ability through direct instruction and scaffolded experience and systemically rewards the corresponding creative outcomes. By doing so, institutions transform from passive arenas into active catalysts that construct the resilient and creative mindset essential for modern challenges.

### 5.3 The moderated mediation effect of student competence

This study also provided significant results to verify the moderated mediation effect of student competence in the first stage of the “achievement motivation → anti- frustration ability → creativity tendency” mediation model. That is, the indirect relationship is moderated by student competence, providing support for H3 and fully addressing the third research question. Specifically, with high student competence, achievement motivated students will establish stronger anti-frustration ability, which further strengthens their creativity tendency. This is because high student competence can play a supporting role in facilitating the impact of achievement motivation.

As a foundational and enduring capacity, student competence enables individuals to navigate their new environment, thereby offering sustained support for their development in higher education (Wiek et al., 2011). Usán et al. (2022) suggests that with highly developed competence, students demonstrate a capacity for positive decision-making, coupled with robust planning and execution abilities, which enables steady progress toward their goals. This study complements findings of prior research that when achievement motivation is present, students with strong competence can apply their decision-making abilities to reduce external uncertainties. They can also utilize their interaction skills to parse the complexities of the situations they are trying to address. Consequently, with this vital asset, motivated students become more confident in their capacity to endure challenging circumstances. This process directly strengthens their anti-frustration ability, thereby improving their tendency for creativity. Conversely, Wu et al. (2023) stated that in the absence of well-developed competence, even if students have strong achievement motivation, they might struggle to analyze encountered issues through methodical analysis, potentially resulting in a failure to identify feasible solutions. This study moves forward to discuss the role of student competence that only with strong motivation but without competence, students cannot solve encountered issues, not mentioning the improvement of anti-frustration ability and subsequent creativity enhancement.

Hence, although there are many potential moderators (e.g., individual personality and activities, and environment) when investigating the relationship between motivational factors and creativity tendency of college students (Correa and Mourao, 2025), because student competence can shape the impact of achievement motivation (Wu et al., 2023), it is incorporated into our model as the first-stage moderator. As such, testing the role of student competence in our model not only enriches the understanding of our proposed mediation effect, but also provides a nuanced understanding to the indirect effect of achievement motivation on creativity tendency.

Our findings thus provide practical implications that in order to enhance students’ creativity, it is necessary to augment their competence in higher education, especially for those with insufficient competence at their enrollment. As an essential supporting capability, student competence is the critical amplifier that facilitate the development of achievement- motivated students for increased creativity via the improvement of their anti-frustration ability. Thus, higher education institutions should take measures to augment student competence, which is the most strategic intervention for unlocking the motivated, resilient, and creative students. For instance, “adaptive challenge problems” can be employed in subjects like engineering or math, in which platforms are adopted to adjust problem difficulty based on a student’s demonstrated competence, ensuring that motivated students are constantly working at the edge of their ability, which builds both their skills and resilience.

### 5.4 Limitations and future research

This study still has some limitations, despite its contributions to the understanding of the relationship and the mechanisms between achievement motivation and creativity tendency in the context of higher education. First, our sample came from fresh students from several Chinese universities. While they represented typical universities in various regions, this sample selection may still encounter the potential issue of generalizability of our findings, given the notable diversity in China’s universities and regions. Future studies may investigate our research questions in alternative research contexts, such as in other provinces, countries or in different types of higher education institutions.

Second, our results provide solid evidence for a partial mediation relationship with anti-frustration ability as the mediator. However, there are alternative mediators, such as self-efficacy. Self-efficacy, defined as a person’s confidence in executing a particular task (Bandura, 1997), can affected by achievement motivation, potentially due to individual pursuit (Li et al., 2023). Students with achievement motivation can be driven by significant personal goals and highly committed, resulting in enhanced self-efficacy. Subsequently, students with high self-efficacy demonstrate a strong capacity for commitment and efforts and exhibit greater persistence in problem-solving when faced with obstacles, leading to greater creativity tendency. Thus, future research should explore other potential mediators to enrich the understanding of the relationship between achievement motivation and creativity tendency.

Third, in this study we included student competence as the first-stage moderator. Nevertheless, there are alternative moderators, such as environmental factors in higher education and teacher characteristics, that may influence the psychological process of college students. As such, future research may consider other contingent situations that can function either in the first or the second stage of the mediation model and thus further enrich our understanding of the focal relationship.

## 6 Conclusion

Aiming to understand the impact of achievement motivation and creativity tendency of college students, this study investigated a moderated mediation model, testing the moderating role in the first stage of the “achievement motivation → anti- frustration ability → creativity tendency” mediation model. Understanding achievement motivation and creativity tendency of college students can be a starting point for fostering their creativity in the process of experiencing higher education. Our findings provide solid evidence to the positive relationship between achievement motivation and creativity tendency. It is also suggested that strengthening achievement motivation of students can improve their anti-frustration ability, which subsequently enhances their creativity tendency. Furthermore, we need to pay attention to student competence, because at different levels of student competence, the impact of achievement motivation on anti-frustration ability differ. These results provide insights into how achievement motivation influences creativity tendency, helping schools design and take measures to cultivate students’ skills and abilities for academic and personal development.

## Data Availability

The original contributions presented in the study are included in the article/Supplementary material, further inquiries can be directed to the corresponding author.

## References

[ref1] Alencar E. M. L. S. Fleith D. S. Pereira N. (2017). Creativity in higher education: challenges and facilitating factors. Temas Psicol. 25, 553–561. doi: 10.9788/tp2017.2-09

[ref2] Alsharari N. M. Alshurideh M. T. (2020). Student retention in higher education: the role of creativity, emotional intelligence and learner autonomy. Int. J. Educ. Manag. 35, 233–247. doi: 10.1108/IJEM-12-2019-0421

[ref3] Amrai K. Motlagh S. E. Zalani H. A. Parhon H. (2011). The relationship between academic motivation and academic achievement students. PRO 15, 399–402. doi: 10.1016/j.sbspro.2011.03.111

[ref4] Atkinson J. W. (1957). Motivational determinants of risk-taking behavior. Psychol. Rev. 64:359. 10.1037/h0043445 13505972

[ref5] Bagozzi R. P. Yi Y. (1988). On the evaluation of structural equations models. J. Acad. Mark. Sci. 16, 74–94.

[ref6] Bandura A. (1997). Self-efficacy: The exercise of control. New York, NY: W. H. Freeman.

[ref7] Baron R. Kenny D. (1986). The moderator–mediator variable distinction in social psychological research: conceptual, strategic, and statistical considerations. J. Pers. Soc. Psychol. 51, 1173–1182. 3806354 10.1037//0022-3514.51.6.1173

[ref9] Block J. Kremen A. M. (1996). IQ and ego-resiliency: conceptual and empirical connections and separateness. J. Pers. Soc. Psychol. 70, 349–361. 8636887 10.1037//0022-3514.70.2.349

[ref9001] Caballero-García P. A. Sanchez-Ruiz S. (2021). Creativity and life satisfaction in Spanish University students. Effects of an emotionally positive and creative program. Front. Psychol. 12, 1–15. 10.3389/fpsyg.2021.746154 PMC8685373 34938232

[ref10] Chen Q. Christensen A. P. Kenett Y. N. Ren Z. Condon D. M. Bilder R. M. . (2023). Mapping the creative personality: a psychometric network analysis of highly creative artists and scientists. Creat. Res. J. 35, 455–470. doi: 10.1080/10400419.2023.2184558

[ref11] Chiu F. C. Hsu C. C. Lin Y. N. Liu C. H. Chen H. C. Lin C. H. (2019). Effects of creative thinking and its personality determinants on negative emotion regulation. Psychol. Rep. 122, 916–943. doi: 10.1177/0033294118775973, 29860928

[ref12] Contractor A. A. Weiss N. H. Forkus S. R. (2021). Moderating effects of dysregulation and fear of positive emotions on the relationship between post-traumatic stress disorder symptoms and positive memory count. J. Clin. Psychol. 77, 701–721. doi: 10.1002/jclp.23046, 32844395 PMC7878328

[ref13] Correa R. Mourao L. (2025). Creativity in higher education: a systematic literature review. Int. J. Educ. Res. 132:102613. doi: 10.1016/j.ijer.2025.102613

[ref14] De Castella K. Byrne D. Covington M. (2013). Unmotivated or motivated to fail? A cross-cultural study of achievement motivation, fear of failure, and student disengagement. J. Educ. Psychol. 105, 861–880. doi: 10.1037/a0032464

[ref15] Deci E. L. Ryan R. M. (1985). “Conceptualizations of intrinsic motivation and self-determination” in Intrinsic motivation and self-determination in human behavior. eds. Deci E. L. Ryan R. M. (New York: Springer), 11–40.

[ref16] Durnali M. Orakci Ş. Khalili T. (2023). Forstering creative thinking skills to burst the effect of emotional intelligence on entrepreneurial skills. Think. Skills Creat. 47:101200. doi: 10.1016/j.tsc.2022.101200

[ref17] Dweck C. (2017). Mindset: The new psychology of success. 1st Edn. Beijing: Random House.

[ref18] Edwards J. R. Lambert L. S. (2007). Methods for integrating moderation and mediation: a general analytical framework using moderated path analysis. Psychol. Methods 12, 1–22. doi: 10.1037/1082-989x.12.1.1, 17402809

[ref19] Egan A. Maguire R. Christophers L. Rooney B. (2017). Developing creativity in higher education for 21st century learners: a protocol for a scoping review. Int. J. Educ. Res. 82, 21–27. doi: 10.1016/j.ijer.2016.12.004

[ref20] Fornell C. Larcker D. F. (1981). Evaluating structural equation models with unobservable variables and measurement error. J. Mark. Res. 18, 39–50.

[ref21] Fredrickson B. L. (2003). The value of positive emotions: the emerging science of positive psychology is coming to understand why it's good to feel good. Am. Sci. 91, 330–335. doi: 10.1511/2003.26.330

[ref9002] Fredrickson B. L. (2013). Positive emotions broaden and build. Adv. Exp. Soc. Psychol. 47, 1–53.

[ref23] Gao T. (2023). College education: problem-solving creativity in an interactive learning environment. Educ. Inf. Technol. 28, 217–236. doi: 10.1007/s10639-022-11150-0

[ref24] Gostoli S. Cerini V. Piolanti A. Rafanelli C. (2017). Creativity, bipolar disorder vulnerability and psychological well-being: a preliminary study. Creat. Res. J. 29, 63–70. doi: 10.1080/10400419.2017.1263511

[ref9003] Guerra N. G. Bradshaw C. P. (2008). “Linking the prevention of problem behaviors and positive youth development: Core competencies for positive youth development and risk prevention,” in Core competencies to prevent problem behaviors and promote positive youth development: New directions for child and adolescent development. Vol. 122. eds. Guerra N. G. Bradshaw C. P.. 1–17. 10.1002/cd.225 19021244

[ref25] Hayes A. F. (2017). Introduction to mediation, moderation, and conditional process analysis: A regression-based approach. New York: Guilford Press.

[ref26] Hewitt P. L. Flett G. L. (1991). Perfectionism in the self and social contexts: conceptualization, assessment, and association with psychopathology. J. Pers. Soc. Psychol. 60:456. 2027080 10.1037//0022-3514.60.3.456

[ref27] Hsiao C. C. (2017). The correlation between elementary pupils’ positive emotion and creativity with creativity tendency and creative self-efficacy as the mediators. J. Educ. Res. Dev. 13, 57–84. doi: 10.3966/181665042017121304003

[ref28] Huang S. L. Zuo H. Mo L. Liu X. Xin T. Lin C. D. (2016). International analysis of core competencies studies for student development. J. Chin. Soc. Educ. 6, 8–14.

[ref29] Jahnke I. Haertel T. Wildt J. (2017). Teachers' conceptions of student creativity in higher education. Innov. Educ. Teach. Int. 54, 87–95. doi: 10.1080/14703297.2015.1088396

[ref30] Kalar B. (2020). The role of creativity in the context of academic entrepreneurship. Creat. Innov. Manag. 29, 254–267. doi: 10.1111/caim.12352

[ref31] Lee H.-W. Liu C.-H. (2009). The relationship among achievement motivation, psychological contract and work attitudes. Soc. Behav. Pers. 37, 321–328. doi: 10.2224/sbp.2009.37.3.321

[ref36] Lin C. (2016). Investigation on the core literacy of students’ development in the 21st century. Beijing: Beijing Normal University Publishing Group.

[ref37] Lin C. (2017). To construct sinicized core competencies and values for student development. J. Beijing Norm. Univ. 1, 66–73. doi: 10.3969/j.issn.1002-0209.2017.01.006

[ref38] Lin H. T. Wang M. R. (1994). Creativity assessment packet. Taipei: Psybooks.

[ref34] Li N. Yang Y. Zhao X. Li Y. (2023). The relationship between achievement motivation and college students’ general self-efficacy: a moderated mediation model. Front. Psychol. 13:1031912. doi: 10.3389/fpsyg.2022.1031912, 36687868 PMC9845271

[ref35] Li Q. Yu K. Gao D. Y. Tang C. X. Liao X. W. Zhang X. D. (2019). College students’ anti-frustration mental ability and dependence on academic frustration: the intermediary function of positive psychological quality. Chin. J. Health Psychol. 7, 1077–1084. doi: 10.13342/j.cnki.cjhp.2019.07.028

[ref39] Livingston L. (2010). Teaching creativity in higher education. Arts Educ. Policy Rev. 111, 59–62. doi: 10.1080/10632910903455884

[ref32] Li X. Long L. R. (2017). The influence of individual characteristics on the choice tendency between teachers and students. J. Psychol. Sci. 40, 1421–1427. doi: 10.16719/j.cnki.1671-6981.20170622

[ref33] Li Y. Wu Q. Li Y. Chen L. Wang X. (2019). Relationships among psychological capital, creative tendency, and job burnout among Chinese nurses. J. Adv. Nurs. 75, 3495–3503. doi: 10.1111/jan.14141, 31241193

[ref40] Lizarraga M. L. S. A. Baquedano M. T. S. A. Closas A. H. (2014). An explanatory model regarding the relationships between psychological traits and creativity. An. Psicol. 30, 355–363. doi: 10.6018/analesps.30.1.153781

[ref41] MacKinnon D. P. Lockwood C. M. Williams J. (2004). Confidence limits for the indirect effect: distribution of the product and resampling methods. Multivar. Behav. Res. 39, 99–128. doi: 10.1207/s15327906mbr3901_4, 20157642 PMC2821115

[ref42] Marczewska M. Weresa M. A. Lachowicz M. (2024). Towards creativity and innovation in universities: study on central and Eastern Europe. J. Knowl. Econ. 15, 1363–1385. doi: 10.1007/s13132-023-01139-6

[ref43] McClelland D. C. (1961). The achieving society. Princeton, NJ: Van Nostrand.

[ref9005] McClelland D. C. (1985). How motives, skills, and values determine what people do. Am. Psychol. 40, 812–825.

[ref44] Mei Y. Cheng K. Liu J. Ye B. (2019). The influence of emotional intelligence on college students’ entrepreneurial intention: the chain mediating effect of achievement motivation and entrepreneurial self-efficacy. Psychol. Explor. 39, 173–178.

[ref45] Moruzzi C. (2021). Measuring creativity: an account of natural and artificial creativity. Eur. J. Philos. Sci. 11, 1–20. doi: 10.1007/s13194-020-00313-w

[ref9006] Neisser U. (1967). Cognitive psychology. Englewood Cliffs, NJ: Prentice Hall.

[ref46] Orakci Ş. (2023). Structural relationship among academic motivation, academic self-efficacy, problem solving skills, creative thinking skills, and critical thinking skills. Psychol. Sch. 60, 2173–2194. doi: 10.1002/pits.22851

[ref47] Ou H. S. Huang Z. J. Zhang X. D. (2013). A study on the effect of anti-frustration mental ability on suicide ideation. Psychol. Explor. 3, 234–238. doi: 10.3969/j.issn.1003-5184.2013.03.008

[ref48] Podsakoff P. M. MacKenzie S. B. Lee J. Y. Podsakoff N. P. (2003). Common method biases in behavioral research: a critical review of the literature and recommended remedies. J. Appl. Psychol. 88, 879–903. doi: 10.1037/0021-9010.88.5.879, 14516251

[ref49] Rampersad G. Patel F. (2014). Creativity as a desirable graduate attribute: implications for curriculum design and employability. Asia-Pac. J. Coop. Educ. 15, 1–11.

[ref50] Rodrigues A. P. Jorge F. E. Pires C. A. Antonio P. (2019). The contribution of emotional intelligence and spirituality in understanding creativity and entrepreneurial intention of higher education students. Educ. Train. 61, 870–894. doi: 10.1108/ET-01-2018-0026

[ref51] Said-Metwaly S. Noortgate W. V. D. Kyndt E. (2017). Approaches to measuring creativity: a systematic literature review. Creativity. Theories – Res. 4, 238–275. doi: 10.1515/ctra-2017-0013

[ref52] Sheridan-Rabideau M. (2010). Creativity repositioned. Arts Educ. Policy Rev. 111, 54–58. doi: 10.1080/10632910903455876

[ref53] Sobel M. E. (1982). Asymptotic confidence intervals for indirect effects in structural equation models. Sociol. Methodol. 13, 290–312.

[ref54] Steinmayr R. Weidinger A. F. Schwinger M. Spinath B. (2019). The importance of students’ motivation for their academic achivement - replicating and extending previous findings. Front. Psychol. 10:1730. doi: 10.3389/fpsyg.2019.01730 31417459 PMC6685139

[ref55] Tugade M. M. Fredrickson B. L. (2004). Resilient individuals use positive emotions to bounce back from negative emotional experiences. J. Pers. Soc. Psychol. 86, 320–333. doi: 10.1037/0022-3514.86.2.320, 14769087 PMC3132556

[ref56] Usán P. Salavera C. Quílez-Robres A. Lozano-Blasco R. (2022). Behaviour patterns between academic motivation, burnout and academic performance in primary school students. Int. J. Environ. Res. Public Health 19:12663. doi: 10.3390/ijerph191912663, 36231963 PMC9566615

[ref9004] Vygotsky L. S. (1978). Mind in society: The development of higher psychological processes. Cambridge, MA: Harvard University Press.

[ref57] Weiss S. Steger D. Kaur Y. Hildebrandt A. Schroeders U. Wilhelm O. (2021). On the trail of creativity: dimensionality of divergent thinking and its relation with cognitive abilities, personality, and insight. Eur. J. Personal. 35, 291–314. doi: 10.1002/per.2288

[ref58] Wiek A. Withycombe L. Redman C. L. (2011). Key competencies in sustainability: a reference framework for academic program development. Sustain. Sci. 6, 203–218. doi: 10.1007/s11625-011-0132-6

[ref59] Williams F. E. (1980). Creativity assessment packet (CAP): Manual. Buffalo: D.O.K. Publishers, Inc.

[ref60] Wu C. (2024). Creative tendency with brain network efficiency: a graph theory analysis. Think. Skills Creat. 53:101556. doi: 10.1016/j.tsc.2024.101556

[ref61] Wu C. (2025). The interaction of online co-creative performance in a paired-player mode with creative tendency as a moderator. Front. Psychol. 16:1388850. doi: 10.3389/fpsyg.2025.1388850, 40927346 PMC12414734

[ref62] Wu M. Huang H. Fu Y. Zhang X. (2023). The effect of anti-frustration ability on academic frustration among Chinese undergraduates: a moderated mediating model. Front. Psychol. 14:1033190. doi: 10.3389/fpsyg.2023.1033190, 36844325 PMC9947130

[ref63] Xu W. Ge S. Ding D. Ren X. (2025). Achievement motivation and performance in wargames: creativity as a mediator. Behav. Sci. 15:557. doi: 10.3390/bs15040557, 40282178 PMC12024209

[ref64] Yam K. C. Christian M. S. Wei W. Liao Z. Nai J. (2018). The mixed blessing of leader sense of humor: examining costs and benefits. Acad. Manag. J. 61, 348–369. doi: 10.5465/amj.2015.1088

[ref65] Yang X. He H. (2018). Developing a scale to measure undergraduates’ antifrustration ability. Soc. Behav. Pers. 46, 633–640. doi: 10.2224/sbp.6555

[ref66] Ye R. Hagtvet K. (1992). Measurement and analysis of achievement motivation. Psychol. Dev. Educ. 8, 14–16.

[ref67] Zhang X. D. (2013). Investigation report on the psychological ability of resisting frustration of college students. Wuhan: Wuhan University Press.

[ref68] Zhang X. D. (2020). Study on coping strategies of academic frustration of college students. Beijing: Chinese Book Company.

