# Supplementary material for: The impact of achievement motivation on creativity tendency of college students: a moderated mediation model
Source: Front Psychol. 2026 Jan 16;16:1720648. doi: 10.3389/fpsyg.2025.1720648 (PMC12855514; doi:10.3389/fpsyg.2025.1720648)
Supplement: Supplementary file 1 [file Data_Sheet_1.pdf]

Supplementary Material

Mixed-effect multinomial logistic regression

We performed a multinomial mixed-effects logistic regression to explore the effect of the social learning condition, the Block of scenario and their interaction on the participants' choice of reaction to each scenario. The DV was made up of 4 different categories (the 4 possible reactions: inaction, gossip, exclusion or confrontation). Therefore, the regression was performed through a set of three category contrasts (Hedeker, 2003) using lme4 (Bates et al., 2015) and lmerTest (Kuznetsova et al., 2017) package in R. As the main goal of the paper is to better understand the use of punishment in response to social norm violations, we decided to use the reaction "do nothing" as reference response for the analyses. In other words, the three punishments were compared to the choice of inaction, allowing us to assess how the probabilities of choosing the other three categories differ relative to choosing to take no action.

In our study, we had a specific expectation regarding the use of punishments within different conditions across blocks of scenarios. We anticipated that the likelihood of using a particular punishment would increase from Block 1 to Block 3. More specifically, we hypothesized the presence of a significant main effect of Block for each type of punishment within their respective condition—seeing others mainly use gossip should increase the probability of choosing "gossip" vs. "inaction". Furthermore, we hypothesized that this effect would be significantly greater for the condition where the observed punishment is the same as the type of punishment being assessed—seeing others mainly use gossip should increase the probability of choosing "gossip" vs. "inaction" more than seeing others mainly use exclusion or confrontation. To test this hypothesis, we aimed to detect significant interaction effects between the block of scenarios and the condition. To run these analyses, we adjusted the reference of the independent variable *Condition* so that it aligned with the punishment being

examined. In practical terms, this means that when we compared the probability of choosing "gossip" to choosing "inaction" as dependent variable, we set the reference for the independent variable *Condition* as the "gossip" condition. Conversely, when comparing the probability of choosing "exclusion" to choosing "inaction " as dependent variable, the reference condition was set to "exclusion" condition. We followed the same logic for the "confrontation" response. This procedure enables us to interpret significant interaction effects as a change between blocks significantly different between the condition group and the two other groups. Negative betas were expected for interaction effects, meaning that the reference group increased the probability of selecting the punishment more importantly.

S1 Table. Reactions seen by participants in the second block according to their experimental condition. G = gossip, E = exclusion, C = confrontation, I = inaction

**trials**	**1**	**2**	**3**	**4**	**5**	**6**	**7**	**8**	**9**	**10**	**11**	**12**	**13**	**14**	**15**	**16**	**17**	**18**	**19**	**20**	**21**	**22**
**Gossip condition**	**G**	I	**G**	**G**	I	E	**G**	**G**	**G**	C	**G**	**G**	C	**G**	**G**	E	**G**	**G**	**G**	I	**G**	**G**
**Exclusion condition**	**E**	I	**E**	**E**	I	G	**E**	**E**	**E**	C	**E**	**E**	C	**E**	**E**	G	**E**	**E**	**E**	I	**E**	**E**
**Confrontation condition**	**C**	I	**C**	**C**	I	G	**C**	**C**	**C**	E	**C**	**C**	E	**C**	**C**	G	**C**	**C**	**C**	I	**C**	**C**
